# A Transcription Factor Signature Can Identify the CMS4 Subtype and Stratify the Prognostic Risk of Colorectal Cancer

**DOI:** 10.3389/fonc.2022.902974

**Published:** 2022-06-30

**Authors:** Min-Er Zhong, Ze-Ping Huang, Xun Wang, Du Cai, Cheng-Hang Li, Feng Gao, Xiao-Jian Wu, Wei Wang

**Affiliations:** ^1^ Department of Colorectal Surgery, The Sixth Affiliated Hospital, Sun Yat-sen University, Guangzhou, China; ^2^ Guangdong Provincial Key Laboratory of Colorectal and Pelvic Floor Diseases, The Sixth Affiliated Hospital, Sun Yat-sen University, Guangzhou, China; ^3^ Department of Biomedical Engineering, School of Basic Medical Science, Central South University, Changsha, China; ^4^ Biomedical Big Data Centre, Department of Gynaecology, Huzhou Maternity & Child Health Care Hospital, Huzhou, China

**Keywords:** colorectal cancer, CMS4, transcription factors, prognosis, prediction, signature

## Abstract

**Background:**

Colorectal cancer (CRC) is a heterogeneous disease, and current classification systems are insufficient for stratifying patients with different risks. This study aims to develop a generalized, individualized prognostic consensus molecular subtype (CMS)-transcription factors (TFs)-based signature that can predict the prognosis of CRC.

**Methods:**

We obtained differentially expressed TF signature and target genes between the CMS4 and other CMS subtypes of CRC from The Cancer Genome Atlas (TCGA) database. A multi-dimensional network inference integrative analysis was conducted to identify the master genes and establish a CMS4-TFs-based signature. For validation, an in-house clinical cohort (n = 351) and another independent public CRC cohort (n = 565) were applied. Gene set enrichment analysis (GSEA) and prediction of immune cell infiltration were performed to interpret the biological significance of the model.

**Results:**

A CMS4-TFs-based signature termed TF-9 that includes nine TF master genes was developed. Patients in the TF-9 high-risk group have significantly worse survival, regardless of clinical characteristics. The TF-9 achieved the highest mean C-index (0.65) compared to all other signatures reported (0.51 to 0.57). Immune infiltration revealed that the microenvironment in the high-risk group was highly immune suppressed, as evidenced by the overexpression of TIM3, CD39, and CD40, suggesting that high-risk patients may not directly benefit from the immune checkpoint inhibitors.

**Conclusions:**

The TF-9 signature allows a more precise categorization of patients with relevant clinical and biological implications, which may be a valuable tool for improving the tailoring of therapeutic interventions in CRC patients.

## Introduction

Colorectal cancer (CRC) is the third most prevalent malignancy worldwide and the second leading cause of cancer-related mortality (1). Even though surgical techniques and perioperative chemotherapy regimens have been vastly improved, the prognosis for patients with CRC remains dismal. The current American Joint Committee on Cancer (AJCC) Tumor, Nodal Involvement, Metastasis (TNM) Staging System (the Eighth Edition) has demonstrated useful but insufficient prediction for prognosis and estimation for different subsets of CRC patients. TNM staging can only describe the anatomical characteristics of the tumor, and it is difficult to reflect the tumor’s inherent heterogeneity and metastatic potential. CRC is a heterogeneous disease with significant differences in survival even among patients with similar clinical characteristics and treatment regimens, indicating that the current classification systems and clinical features are insufficient to stratify patients with different risks effectively. Increasing evidence suggests that the development and application of effective molecular biomarkers could facilitate the prognostic assessment and identification of potential cancer patients at high-risk (2–4). With the advancement of sequencing technology and the availability of large-scale public cohorts with gene expression data, a more generalized biological background-based prognostic signature can be identified.

Cancer initiation and progression have been associated with transcription factors (TFs) (5, 6). TFs are proteins that bind to DNA-regulatory sequences (enhancers and silencers), which could potentially regulate gene expression and protein synthesis. In other words, the function of TFs is to activate or inhibit the transcription of specific genes, thus being the primary determinant of the gene function at a given time.

Recently, the consensus molecular subtypes (CMSs) groups were considered the most reliable classification system available for CRC (7). This classification system divides CRC into four subtypes with distinguishing characteristics. CMS4 is the mesenchymal type characterized by the prominent activation of transforming growth factor-β, stromal invasion, and angiogenesis (7). Notably, among the four CMSs, CMS4 has the lowest survival rate. Previous studies on breast cancer have demonstrated that subtype-specific prognostic signatures can significantly improve risk stratification, which may lead to more precise treatment for patients (3). Consequently, present studies are more focusing on the most invasive CMS4 subtype and conducting network inference by integrating the differentially expressed TF signature and target genes between the CMS4 and other CMS subtypes.

This study analyzed the genomic data of more than 1000 CRC patients from three cohorts. Through multi-dimensional network analysis, we identified the dominant TF signature that regulates the most aggressive CRC subtype, CMS4. TF-9, a nine-gene signature, was developed and validated in two additional validation cohorts. According to our study, TF-9 is identified as a potential risk stratification classifier and may serve as a predictor of the response to chemotherapy and immune checkpoint immunotherapy.

## Materials and Methods

### Public Data Source

A total of 1537 CRC patients from three independent cohorts were included in the current study. We obtained 351 CRC samples from our in-house database and 1186 samples from two publicly available datasets. The TCGA dataset (n = 621) (8) was set as the training cohort. GSE39582 (n = 565) (9) and the in-house cohort (n = 351) were used as validation cohorts. TCGA datasets were downloaded by the “TCGAbiolinks” package (version 2.18.0) (10). The normalized expression profiling and corresponding clinical data of GSE39582 were collected from the Gene Expression Omnibus (GEO) by using the “GEOquery” package (version 2.56.0) (11). The clinical characteristics of the patients included in the current study are summarized in **Supplementary Table S1**.

### In-House Clinical Cohort

The in-house cohort is one of the colorectal cancer subprojects of the International Cancer Genome Consortium-Accelerate Research in Genomic Oncology (ICGC-ARGO) project (https://www.icgc-argo.org/). The normalized RNA expression matrix and clinical data for this cohort were obtained from our center. This study was approved by the Medical Ethics Committee of the Sixth Affiliated Hospital of Sun Yat-sen University.

### Integrated Network Analysis

The procedure is depicted schematically in **Supplementary Figure S1**. In brief, 1589 transcription factor (TF) signature genes were retrieved from Lambert’s (5). Univariate Cox was applied to identify TF genes linked to overall survival (OS). The TF genes measured across all datasets were evaluated. By integrating the differentially expressed molecular modalities and TF genes within the CMS4 subtype, we inferred the relationship between TF signals and potential target genes. The limma package (version 3.42.2) (12) in R was utilized to analyze the differential expression of TF genes and potential target genes between the CMS4 and other CMSs. Differentially expressed TF genes were identified when log2FC > 0.5 and adjusted P < 0.05. Target genes were identified as differentially expressed when log2FC > 1.25 and adjusted P < 0.05. Using the TCGA cohort as training data, the RTN package (version 2.10.0) (13) was used to conduct network inference analysis. More specifically, the network analysis incorporates three steps: firstly, compute mutual information between a TF gene and all potential targets, removing non-significant associations by permutation analysis; secondly, remove unstable interactions by bootstrapping; and finally, apply the ARACNE (Algorithm for the Reconstruction of Accurate Cellular Networks) (14) algorithm to reduce redundant indirect regulations. Master regulator analysis (MRA) was performed to examine the overrepresentation of the CMS4 signature in the regulation of each TF gene by hypergeometric testing.

### Development and Evaluation of the TF Signature for CRC

After the hypergeometric tests resulted for all TF genes, adjusted p-values were calculated using the Benjamini-Hochberg procedure. Nine TF genes were identified as master regulatory factors and were significantly upregulated in CMS4. The TF-9 prognostic signature was developed using the multivariable Cox regression model in the TCGA cohort with these nine signature genes. The risk score formula was constructed based on a linear combination of the expression levels weighted with the regression coefficients: TF-9 = (-0.1582×MEIS3) + (0.131×SNAI1) + (0.0253×KLF17) + (0.0841×BARX1) + (-0.031×ZNF532) + (0.3504×HEYL) + (0.0872×FOXL2) + (-0.0267×LHX6) + (0.0789×MEIS2). Risk scores were calculated for all patients in the TCGA cohort and the two validation cohorts. Based on the median score of each cohort, patients were divided into high-risk and low-risk subgroups. The prognostic relevance of TF-9 was further evaluated using Kaplan-Meier survival analysis on two independent validation datasets.

### Gene Set Enrichment Analysis (GSEA) and Immune Cell Infiltration Prediction by CIBERSORT

GSEA was performed using the HTSanalyzeR package (version 2.3.5) (15). Gene sets data were downloaded from the Molecular Signatures Database (MSigDB, https://www.gsea-msigdb.org/gsea/msigdb/) (16). To evaluate the immunobiological difference between the high-risk and low-risk groups, CIBERSORT was used to characterize 22 types of immune cells’ abundance for each sample. Specifically, standardized gene expression series were uploaded to the CIBERSORT portal (http://cibersort.stanford.edu/) with 1,000 permutations.

### Survival Analysis

Using the Kaplan-Meier method, the OS and recurrence-free survival (RFS) rates were calculated for all three cohorts. The log-rank test was utilized to compare the survival curves of the patients in the high- and low-risk groups.

### Comparison With Existing Classifiers

We calculated the signature scores of Lee’s (17), Ren’s (18), and Ye’s (19) by re-building multivariable Cox proportional-hazards models using the TCGA and ICGC-ARGO datasets with the published classifier genes, respectively. We calculated the concordance index (C-index) and the robust hazard ratio (D-index) for the three previous classifiers and TF-9 using TCGA and ICGC-ARGO cohorts by the survcomp package (version 1.42.0) (20).

### Statistical Analysis

Statistical analyses were performed with the R program (version 3.6.1, R Foundation for Statistical Computing, Vienna, Austria. http://www.R-project.org/). A Univariate COX proportional hazards model was used to investigate the prognostic value of the selected TF signature. Univariate and multivariate Cox regression analyses were done to identify the independent prognostic effect of TF-9. The Student’s t-test was applied to assess the nine TF signature genes and risk score distribution in different conditions. The Pearson correlation was performed to reveal correlations between the TF-9 scores with epithelial-mesenchymal transition (EMT) signature genes. The receiver-operating characteristic (ROC) analysis was performed to evaluate the specificity and sensitivity of TF-9 in identifying the CMS4 subtype. The Kaplan-Meier method was used to analyze survival. The Benjamini–Hochberg procedure was applied to control the false discovery rate (FDR). Unless otherwise specified, a two-sided P-value < 0.05 was considered statistically significant.

## Results

### Multi-Dimensional Network Inference Integrative Analysis Identified Nine TF Genes as Key Regulators in the CMS4 Subtype

**Figure 1:**
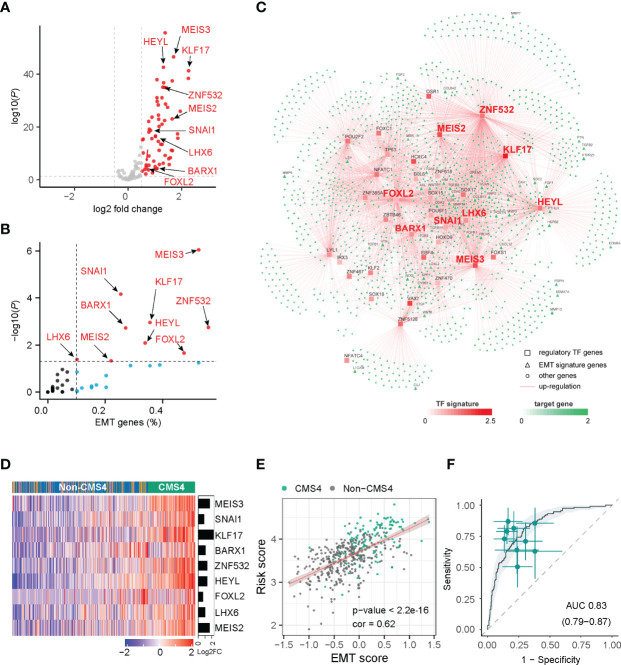
Network inference identified nine transcription factors gene as key regulators of CMS4 subtype in colorectal cancer. **(A)** Volcano plot of the differentially expressed genes in the CMS4 vs. other CMS subtype and highlighting the nine candidate transcription factors genes. **(B)** All these nine transcription factor genes can regulate EMT genes. **(C)** Integrated network showing the relationships between the expression profile of nine transcription factor genes and target genes. **(D)** Heatmap of the expression of nine candidate transcription factors-related genes in CMS4 and other CMSs. **(E)** Correlation analysis demonstrated a positive correlation between the TF-9 signature risk score and EMT score (correlation coefficient = 0.62, P < 0.001). **(F)** TF-9 can distinguish CMS4 from other CMS subtypes of colorectal cancer, with AUC values of 0.83.

Previously, the CMS4 subtype of CRC was characterized by the poorest survival rate among the four CMSs (21–23). Our results are consistent with it. The CMS4 subtype presented the worst outcomes compared to other CMSs in TCGA (**Supplementary Figure S2**). By focusing on the CMS4 subtype, we investigate the regulatory role of TFs in CRC by multi-dimensional network inference integrative analysis. A total of 1537 cases from three independent datasets were enrolled in our analysis (**Supplementary Table S1**). And 1589 transcription factor-related genes (TF genes) were downloaded from Lambert’s study (5). After univariate Cox analysis, 116 TF genes were identified to be correlated with CRC OS. Based on the TCGA cohort, we performed a differential analysis of TF genes and potential target genes between the CMS4 subtype and other CMSs. As a result, 62 TF genes (log2 FC > 0.5, BH-adjusted P < 0.05) and 1693 target genes (log2 FC > 1.25, BH-adjusted P < 0.05) were identified as differentially expressed genes in CMS4 of CRC (**Figure 1A**).

With the expression profiles of these preferential TFs and their target genes, an intricate regulatory network was developed by calculating the mutual information between a TF signature and its potential targets. Nine TF-related genes (*MEIS3*, *SNAI1*, *KLF17*, *BARX1*, *ZNF532*, *HEYL*, *FOXL2*, *LHX6*, *MEIS2*) were identified as core regulators for the CMS4 subtype (**Figures 1A–C**, **Supplementary Table S3**) by master regulator analysis (MRA). MRA demonstrated that all nine of these TF genes were EMT genes (**Figure 1B**). Compared with other CMS subtypes, these nine candidate TF genes were significantly upregulated in the CMS4 subtype (**Figure 1D**, **Supplementary Figure S3**). According to the microsatellite instability (MSI) status, HEYL and SNAI1 were downregulated in MSI patients, FOXL2 was upregulated, and the other six genes were not significantly different (**Supplementary Figure S4**).

### Development of the TF-9 Signature

The risk model termed TF-9 was constructed with the coefficients generated from the multivariable Cox proportional-hazards model. After extracting coefficients from the results, we calculated risk scores with coefficient-weighted expression levels of these nine TFs: risk score = (-0.1582×*MEIS3*) + (0.131×*SNAI1*) + (0.0253×*KLF17*) + (0.0841×*BARX1*) + (-0.031×*ZNF532*) + (0.3504×*HEYL*) + (0.0872×*FOXL2*) + (-0.0267×*LHX6*) + (0.0789×*MEIS2*). As CMS4 tumors exhibited high overexpression of genes associated with EMT (7), correlation analysis was performed between the TF-9 risk score and EMT score for further investigation. Unsurprisingly, the TF-9 risk score exhibited a substantial positive correlation with the EMT score (correlation coefficient = 0.62, P < 0.001, **Figure 1E**), indicating that the EMT may be regulated by these nine genes. These results suggest that these nine genes were the master genes that regulated the CMS4 subtype, and the TF-9 was highly related to EMT. Because the calculation of CMS classification relies on the sequencing information of tumors, its clinical translation and application are hampered. Additionally, since these nine TF genes are the master genes that regulate the CMS4 subtype, we wondered whether TF-9 could be a tool for identifying CMS4. Therefore, the ROC curve was performed to examine the performance of TF-9 as a biomarker for identifying CMS4. The result shows that the diagnostic performance of TF-9 for distinguishing CMS4 was satisfactory, with an AUC value of 0.83 in the TCGA cohort (**Figure 1F**). The same performance was achieved in two independent validation cohorts, with AUC values of 0.86 for GSE39582 and 0.89 for ICGC-ARGO (**Supplementary Figure S5**).

### TF-9 Can Predict the Outcome of CRC Patients

**Figure 2:**
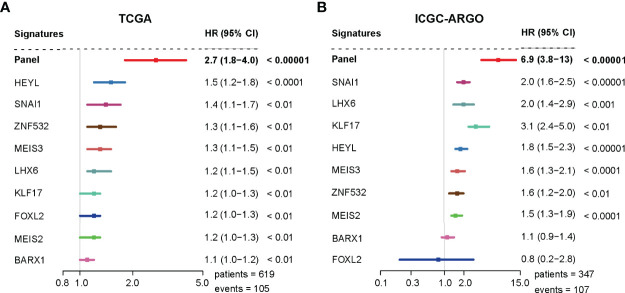
The TF-9 signature is implicated as an independent prognostic factor in CRC. **(A)** Both the TF-9 signature and all nine candidate transcription factors show prognostic value in the TCGA cohort, while the TF-9 with a more significant HR (HR = 2.7, 95%CI:1.8-4.0, P < 0.001). **(B)** Both the TF-9 signature and all nine candidate transcription factors show prognostic value in the ICGC-ARGO cohort, while the TF-9 with a more significant HR (HR = 6.9, 95%CI: 3.8-13.0, P < 0.001).

**Figure 3:**
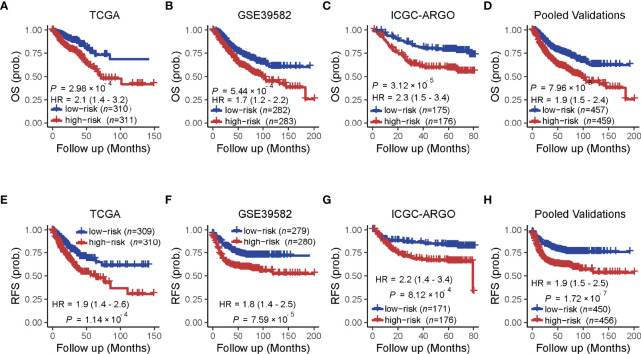
Prognostic value of the TF-9 for colorectal cancer. **(A)** Kaplan–Meier survival analysis showed that the high-risk group had worse overall survival than the low-risk group in the training cohort (TCGA). **(B, C)** In the two independent validation cohorts and **(D)** the pooled cohorts, the high-risk group also showed a significantly poor prognosis for overall survival. **(E–H)** The training cohort, the two independent validation cohorts, and the pooled validation cohort demonstrated that the high-risk group showed a significantly poor prognosis for recurrence-free survival. P-values were calculated by log-rank tests. “OS” refers to overall survival; “RFS” refers to recurrence-free survival.

**Figure 4:**
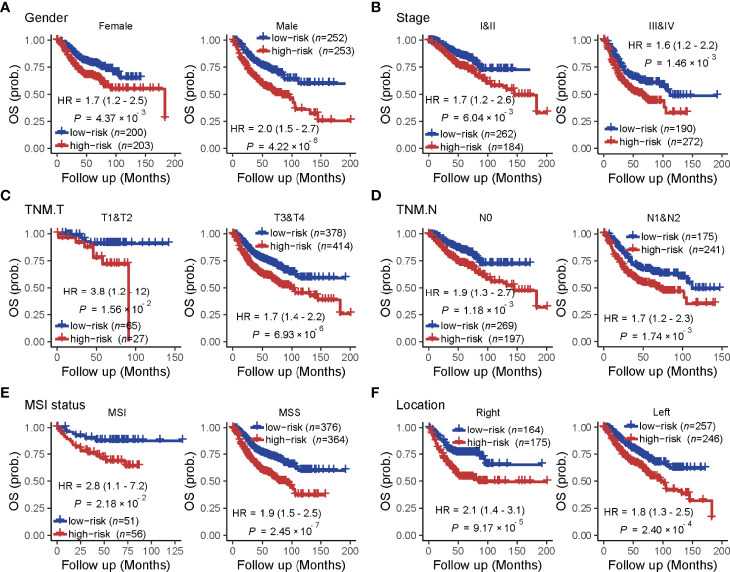
The prognostic value of the TF-9 in colorectal cancer is stratified by clinical characteristics. Even stratified by **(A)** gender, **(B)** stage (I&II vs. III &IV), **(C)** T stage (T1&T2 vs. T3 &T4), **(D)** N stage (N0 vs. N1&N2), **(E)** MSI status (MSI vs. MSS) and **(F)** primary tumor location (right-sided vs. left-sided), TF-9 can still stratify patients into low- and high- risk groups with significant prognosis value.

A univariate analysis was performed to evaluate the prognostic potential of these nine TF genes. As shown in **Figure 2**, the expression of these nine TF genes was implicated as independent prognostic factors in CRC in both the TCGA and ICGC-ARGO cohorts. To further investigate the prognostic value of the TF-9 signature, the risk score of TF-9 was calculated for patients in the TCGA and ICGC-ARGO cohorts. As a result, the TF-9 showed prognostic efficiency with an obvious higher HR in the TCGA (HR = 2.7, P < 0.001) and ICGC-ARGO (HR = 6.3, P < 0.001) cohorts (**Figure 2**). Then, all patients were divided into TF-9 low- and high-risk groups by the median risk value within each cohort (**Supplementary Table S4**). Survival analysis revealed that CRC patients with TF-9 high-risk showed significantly worse OS than patients in the low-risk group in the training cohort (**Figure 3A**; HR = 1.7, P = 1.46×10^-2^). Moreover, the high-risk group showed significantly reduced OS compared with the low-risk group in two validation cohorts (**Figures 3B,C**). A more significant survival diversity was observed between the high- and low-risk groups in the pooled validation datasets (**Figure 3D**). Since tumor recurrence plays a vital role in the poor prognosis of CRC, we also performed survival analyses focusing on RFS. As demonstrated in **Figures 3E–H**, the risk score of TF-9 was also negatively correlated with RFS. In addition, the TF-9 remains effective at discriminating survival after adjusting to clinical factors associated with prognosis, including gender, TNM stage, MSI status (MSI vs. MSS), and primary tumor location (left- vs. right-sided, **Figure 4**). Even when stratified by mutation of RAS or APC, TF-9 can still stratify patients into low- and high-risk groups with significant prognosis value (**Supplementary Figure S6**). Unsurprisingly, both univariate and multivariate Cox analyses identified the TF-9 signature as an independent prognostic factor for CRC (**Supplementary Table S2**).

### TF-9 Shows Its Superiority in Prognostic Prediction Compared With Existing Models

**Figure 5:**
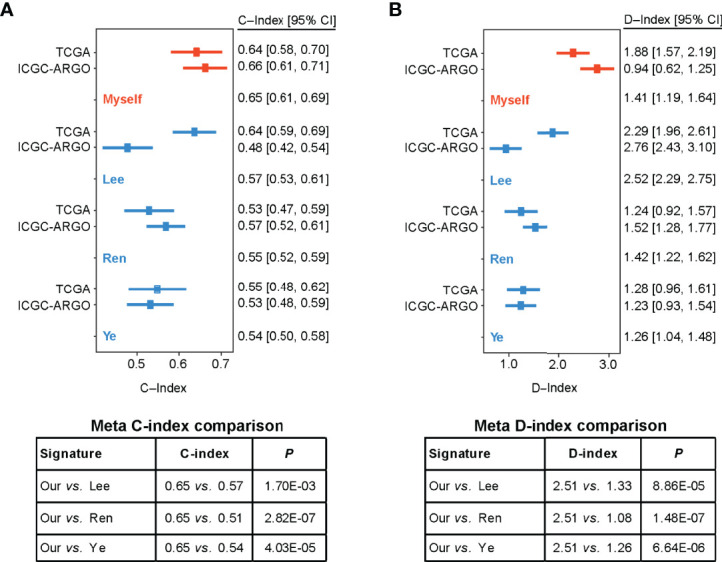
Forest plot reporting of C-index and D-index of various prognostic signatures among the different cohorts. **(A)** The concordance indices (C-index) for TCGA and ICGC-ARGO cohorts. Our model achieved the highest C-index compared to the three reported models (0.65 vs. 0.51-0.57). **(B)** The robust hazard ratio (D-index) for TCGA and ICGC-ARGO cohorts. Our model achieves the highest D-index compared to the three reported models (2.51 vs. 1.08-0.33).

To compare the prognostic value of the TF-9 gene signature with existing prognostic classifiers, the C-index and D-index were calculated with survival data from the TCGA and ICGC-ARGO cohorts. The C-index was significantly higher in TF-9 than in the existing Lee, Ren, and Ye prognostic systems (Meta C-index, TF-9 vs. Lee: 0.65 vs. 0.57, P < 0.01; TF-9 vs. Ren: 0.65 vs. 0.51, P < 0.01; TF-9 vs. Ye: 0.65 vs. 0.54, P < 0.01; **Figure 5A**). Similar to the C-index, the D-index of TF-9 was also significantly higher in TF-9 than in Ren, Lee, and Ye’s prognostic systems (Meta D-index, TF-9 vs. Lee: 2.51 vs. 1.33, P < 0.01; TF-9 vs. Ren: 2.51 vs. 1.08, P < 0.01; TF-9 vs. Ye: 2.51 vs. 1.26, P < 0.01; **Figure 5B**). The above results showed the potential and robustness of TF-9 as a prognostic prediction platform.

### Functional Analysis Reveals the Characteristic Pathway of CMS4

**Figure 6:**
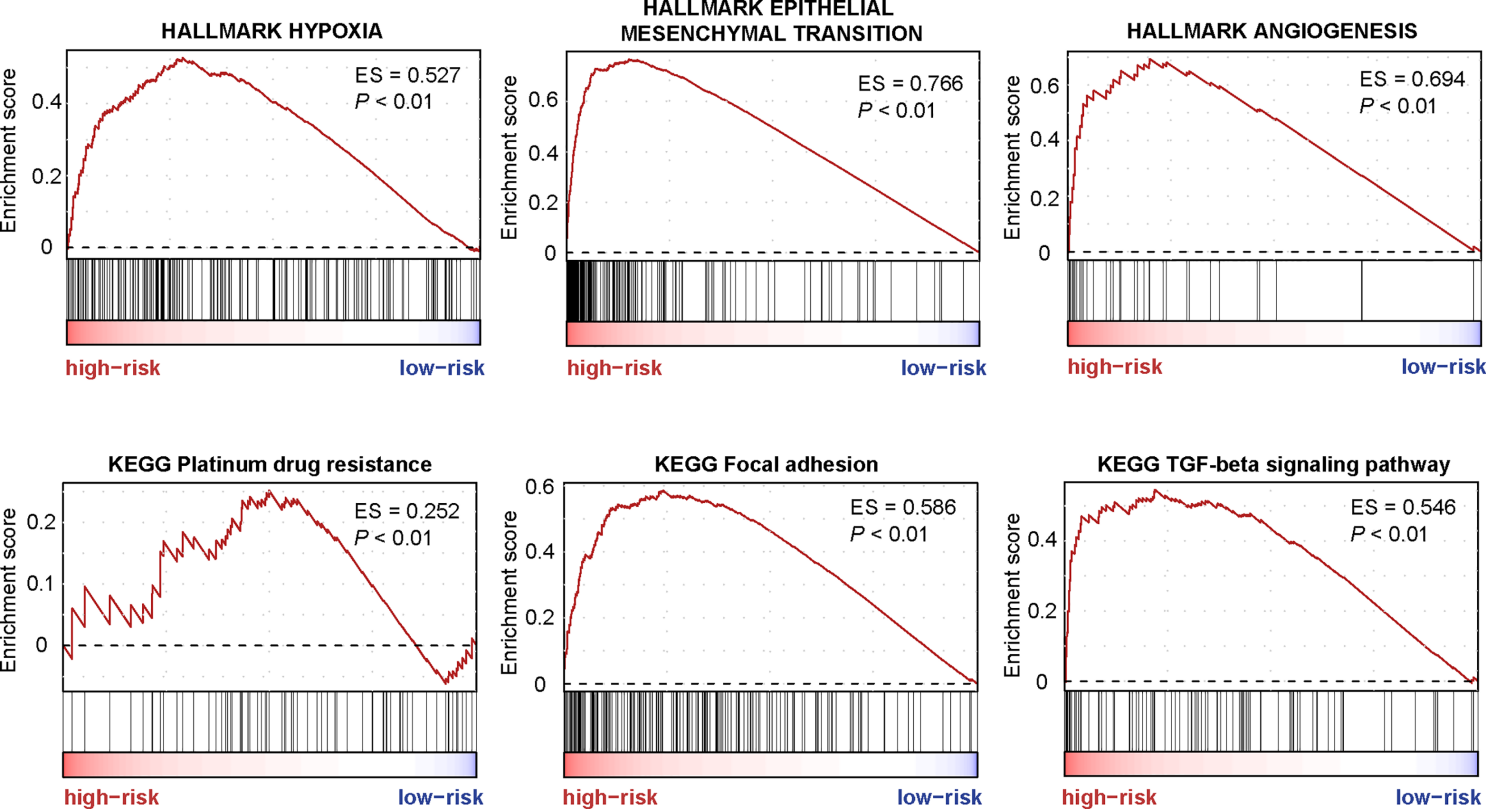
The enriched pathways are associated with the TF-9 signature. GSEA revealed that these nine transcription factor genes are mainly related to hallmark gene sets of EMT, hypoxia, angiogenesis, Platinum drug resistance, focal adhesion, and the TGF-beta signaling pathway.

GSEA was performed to screen the differently enriched pathways between the high- and low-risk groups according to the TF-9 signature. As a result, 131 gene sets (P < 0.01) were upregulated, and 39 gene sets (P < 0.01) were downregulated in the high-risk group (**Supplementary Table S5**). GSEA revealed that these nine TF genes are mainly related to hallmark gene sets of EMT, hypoxia, angiogenesis (**Figure 6**), and KRAS signaling up (**Supplementary Figure S7**). Moreover, TF-9 high-risk groups are enriched in KEGG pathways closely associated with clinical treatment effects such as platinum drug resistance, focal adhesion, TGF-beta signaling pathway (**Figure 6**), and Wnt signaling pathway (**Supplementary Figure S7**). The functional analysis indicates that drugs targeting the oncogenic pathway may have different efficacy in the high- and low-risk groups. The TF-9 may help to stratify CRC patients to explore new target regimens.

### Genomic Omics Reveal the Immune-Suppressed Status in the TF-9 High-Risk Group

**Figure 7:**
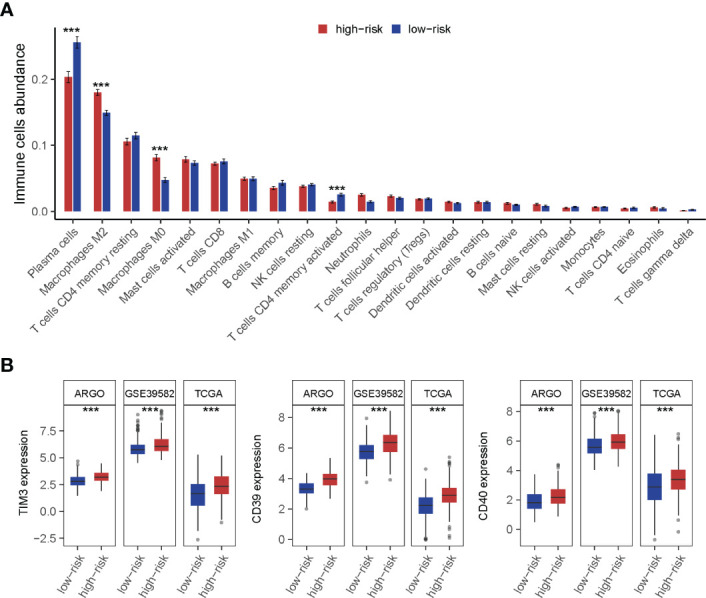
Immune cell infiltration analysis of high-risk and low-risk groups. **(A)** The infiltration of 22 types of immune cells’ abundance for high- and low-risk groups. The high-risk group was associated with decreased densities of plasma cells and CD4 memory-activated T cells. And low-risk patients tended to be infiltrated with fewer M2 and M0 macrophages, while no significant difference was found within other immune cell types. **(B)** TIM3, CD39, and CD40 were upregulated in the high-risk group. ***P < 0.001

The landscape of CRC infiltrating immune cells has not been fully elucidated. We investigated immune infiltration of TF-9 high-risk and low-risk groups in 22 subpopulations of immune cells using the CIBERSORT algorithm. Of note, the high-risk group was associated with decreased densities of plasma cells and CD4 memory-activated T cells. Low-risk patients tended to be infiltrated with fewer M2 and M0 macrophages, while no significant difference was found within other immune cell types (**Figure 7A**). Together, it revealed the immune-suppressed status in the high-risk group. To explore the potential mechanism of immune suppression, the expression of various immune checkpoints was calculated in each group. Surprisingly, in three independent cohorts, upregulated TIM3, CD39, and CD40 were observed in the high-risk group (**Figure 7B**).

## Discussion

We found nine TF genes as the key regulators affecting the progression of the CMS4 subtype in CRC. *MEIS2* and *MEIS3* are members of the MEIS family. Some studies identified the MEIS family as oncogenes, while others recognized them as tumor suppressor genes (24–26). It was reported that *MEIS2* promotes cell migration and invasion in CRC (27). And *MEIS3* can modify the sensitivity to cetuximab *via* c-Met and Akt (28). Overexpression of *SNAI1* sustains stemness maintenance and promotes invasion in numerous cancers, including CRC (29, 30). *KLF17* was considered a favorable prognosis biomarker since it suppresses EMT and metastasis (31, 32). *BARX1* was hypermethylated in some patients with CRC (33), and its expression was a predictor of relapse-free survival for gastrointestinal stromal tumors (34). *ZNF532* has been linked to the prognosis of pancreatic ductal adenocarcinoma (35). *HEYL* modulates the metastasis forming capacity of CRC (36). *FOXL2* regulates a range of target genes related to genomic integrity and cell pathways, including cell cycle progression, proliferation, and apoptosis (37, 38). Previous studies have shown that *LHX6* can play a tumor inhibitory role by inhibiting the downstream genes related to cell proliferation, cell migration, and metastasis (39).

On the basis of these nine genes, we developed a CRC prognostic model termed TF-9. TF-9 shows strong robustness in prognostic risk stratification, regardless of whether it is applied to public data or in-house cohorts. Patients in the high-risk group had a lower survival rate, regardless of OS or RFS, and this is irrespective of clinical characteristics such as gender, stage, MSI status, and primary tumor location. Meanwhile, the C index and D index demonstrated that TF-9 is superior to existing prognostic models. We anticipate that TF-9 will considerably contribute to the stratification of patients with CRC as a robust prognostic prediction model. It is worth mentioning that the TF-9 signature may reliably identify CMS4 based just on the expression of nine TF genes without the need for comprehensive sequencing information. This is of great significance for decreasing the cost of CMS classification in clinical practice.

Moreover, through bioinformatics analysis and functional annotation of these nine TF genes, we believe that TF-9 is also helpful in explaining the biological behavior of high-risk CRC and predicting drug sensitivity. It’s well known that the CMS4 is characterized as a mesenchymal phenotype with hallmark features including EMT, angiogenesis, integrin upregulation, and stromal infiltration (40). Consistent with it, all these nine TF genes are significantly upregulated in CMS4 and are positively correlated with EMT. Moreover, the dysregulated genes of high-risk patients stratified by TF-9 risk score were found to be enriched in tumor-related signaling pathways such as EMT, angiogenesis, hypoxia, TGF-beta signaling, and platinum drug resistance pathways. These results once again confirm the mesenchymal phenotype of the CMS4 subtype and suggest that there may be different chemotherapy sensitivities between the high- and low-risk groups. GSEA revealed that the high-risk group was significantly enriched in the platinum drug resistance pathway. It is inferred that CRC patients in the low-risk group may be more sensitive to chemotherapy regimens based on platinum drugs. The FIRE3 (AIO KRK-0306) trial demonstrated that CMS4 possibly benefits more from anti-EGFR than anti-VEGF therapy. Within the RAS wild-type patients, OS observed in CMS4 favored FOLFIRI cetuximab over FOLFIRI bevacizumab (41). Combined with the results of our study, CMS4 can also be subdivided into two diverse risk subgroups. Inferring from the differential enrichment of EMT and angiogenesis pathways in the two risk groups, the efficacy of the two groups on anti-EGFR therapy may be completely different. Perhaps the high-risk group can benefit from anti-VEGF treatment, while the low-risk group will benefit more from anti-EGFR treatment. It is exciting and needs to be explored in further clinical trials.

Except for cytotoxic chemotherapy and targeted therapy, immune checkpoint inhibitors (ICIs) targeting programmed cell death protein 1 (PD-1) or PD-1 ligand (PD-L1) have emerged as promising treatment strategies in CRC that lead to durable antitumor activities and improved survival (42, 43). However, not all CRCs have ICIs indications. Our analysis of the patients’ immune infiltration revealed that the microenvironment in the high-risk group presented highly immunosuppressed characterized by TIM3, CD39, and CD40 overexpression, which indicated the patients in the high-risk group might not benefit from traditional ICIs such as anti-PD1 therapies directly. Nevertheless, these three immunosuppressive molecules may suggest new therapeutic targets or regimens for patients in the high-risk group.

TIM3 is a negative immune checkpoint and makes a crucial contribution to tumor-induced immune suppression. Accumulating evidence shows that high levels of TIM3 expression correlate with T cell exhaustion and inferior clinical outcomes of cancers (44–46). TIM3 expression in patients’ lymphocytes has been implicated in resistance to immune checkpoint blockade, representing a potential novel target for cancer immunotherapy (47). High levels of CD39 have been associated with advanced grade or poor disease outcomes in multiple malignancies (48–50). Existing studies have shown inhibition of CD39 activity can restore the sensitivity of autophagy-deficient tumors to immunogenic chemotherapy (51). It has been reported that combining anti-PD1/PDL1 with CD39 inhibition results in a synergistic effect. When anti-PD1 therapy was combined with CD39 enzymatic inhibition, it demonstrated significant tumor growth inhibition in mice with tumors refractory to immunotherapy (52). In other words, anti-PD1 treatment combined with CD39 inhibition may sensitize the TF-9 high-risk CRC tumor to immune checkpoint blockade. CD40 is a cell-surface member of the TNF (tumor necrosis factor) receptor superfamily. Upon activation, CD40 can turn tumors from the immune “cold” state to the immune “hot” ones (53), sensitizing them to checkpoint inhibition. In short, these three immunosuppressive molecules mentioned above may all become new targets for immunotherapy in TF-9 high-risk patients, which needs to be further investigated.

We have perceived several limitations in this study. Firstly, although this study included an in-house cohort and independent external validations, it was difficult to avoid missing information when data were retrospectively collected in publicly available databases. Secondly, it is difficult to perform original quality control on public datasets. Thirdly, although we showed the enriched pathway and complex immune microenvironment between high- and low-risk groups, we lack the experiments to confirm this finding *in vivo* and *in vitro*. Therefore, the findings of this study need to be further verified by a well-designed, prospective, multi-center study.

## Conclusions

The TF genes are associated with the prognosis of CRC patients and can identify the CMS4 subtype from other CMSs. The TF-9 signature allows a more precise categorization of patients with relevant clinical and biological implications, which may be valuable tools to improve tailored therapeutic interventions in CRC patients.

## Data Availability Statement

Publicly available datasets were analyzed in this study. This data can be found here: https://www.ncbi.nlm.nih.gov/geo/; https://portal.gdc.cancer.gov/.

## Ethics Statement

Ethical review and approval was not required for the study on human participants in accordance with the local legislation and institutional requirements. Written informed consent for participation was not required for this study in accordance with the national legislation and the institutional requirements.

## Author Contributions

Conception, design, and drafting of the article: WW, X-JW, and FG. Development of methodology: WW, X-JW, and FG. Acquisition of data: M-EZ, Z-PH, XW, DC, and C-HL. Analysis and interpretation of data: M-EZ, Z-PH, and XW. Writing, review, and/or revision of the manuscript: M-EZ, Z-PH, and XW. Administrative and technical support: WW, X-JW, and FG. Verified the underlying data: WW and FG. All authors have read and approved the final version of the manuscript.

## Funding

This work was supported by the National Natural Science Foundation of China (82002221, 82102475) and the China Postdoctoral Science Foundation (2020M683121, 2021T140769). This work was supported by National Key Clinical Discipline.

## Conflict of Interest

The authors declare that the research was conducted in the absence of any commercial or financial relationships that could be construed as a potential conflict of interest.

## Publisher’s Note

All claims expressed in this article are solely those of the authors and do not necessarily represent those of their affiliated organizations, or those of the publisher, the editors and the reviewers. Any product that may be evaluated in this article, or claim that may be made by its manufacturer, is not guaranteed or endorsed by the publisher.

## Abbreviations

CMS, consensus molecular subtype; TFs, Transcription factors; CRC, Colorectal cancer; AJCC, American Joint Committee on Cancer; TF-9, Nine transcription factor related genes’ signature; TCGA, The Cancer Genome Atlas; GEO, Gene Expression Omnibus; MRA, Master regulator analysis; GSEA, Gene set enrichment analysis; OS, Overall survival; RFS, Recurrence-free survival; TF genes, Transcription factor-related genes; C-index, concordance index; D-index, robust hazard ratio; EMT, Epithelial-mesenchymal transition; ROC, Receiver-operating characteristic; FDR, False discovery rate; MSI, microsatellite instability; ICI, immune checkpoint inhibitor; PD1, programmed cell death protein 1; PD-L1, programmed cell death protein 1 ligand; dMMR, mismatch repair deficiency; MSI-H, microsatellite instability-high; TILs, tumor-infiltrating lymphocytes; TNF, tumor necrosis factor.
