# Supplementary material for: A Transcription Factor Signature Can Identify the CMS4 Subtype and Stratify the Prognostic Risk of Colorectal Cancer
Source: Front Oncol. 2022 Jun 30;12:902974. doi: 10.3389/fonc.2022.902974 (PMC9280271; doi:10.3389/fonc.2022.902974)
Supplement: Supplementary file 1 [file DataSheet_1.pdf]

Supplementary Materials

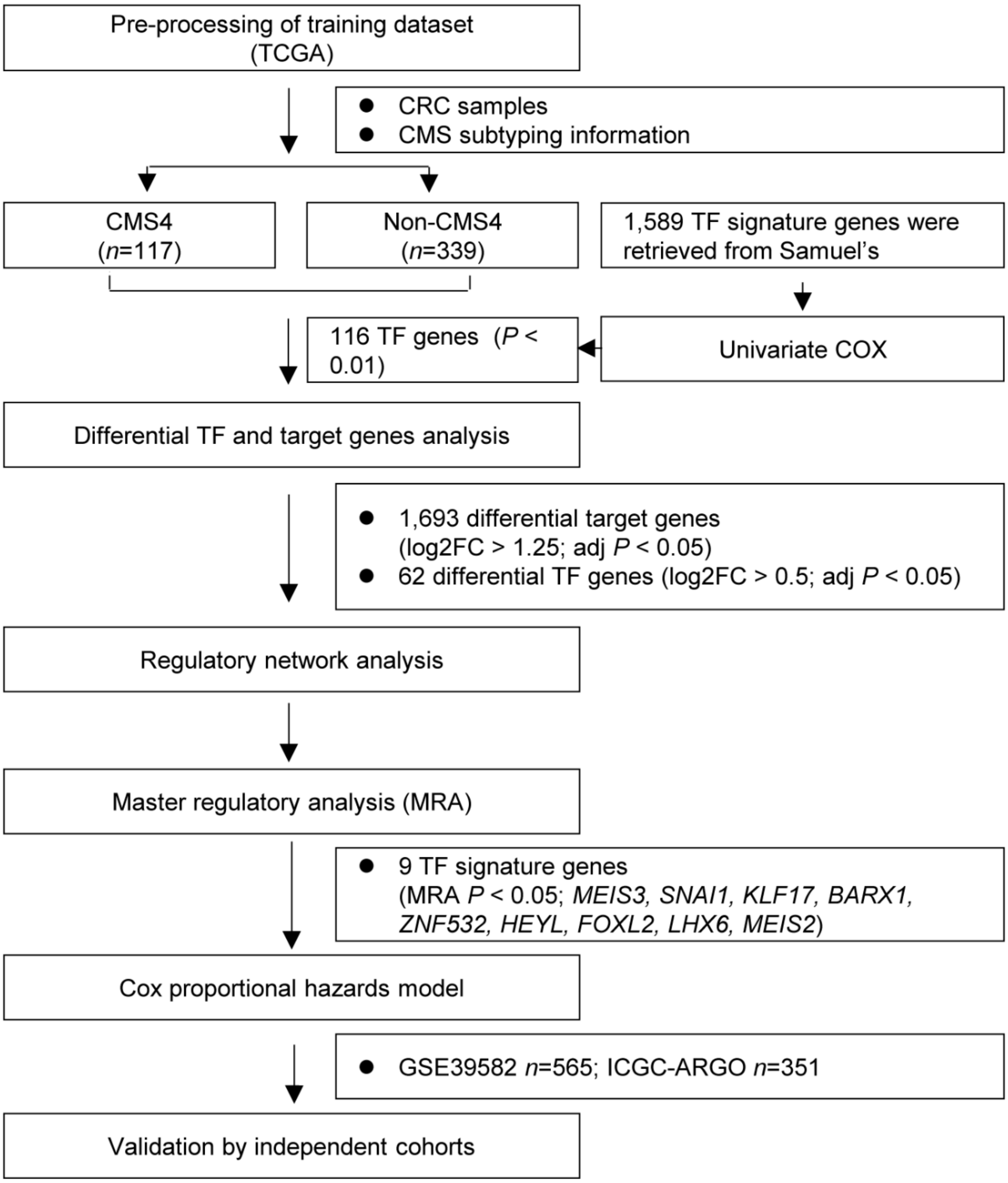

Supplementary Figure 1. Schematic workflow. Two public colorectal cancer datasets and one in-house cohort containing 1,537 cases were included in the current study. A nine-gene transcription factors signature was developed in the training set and validated in two independent cohorts.

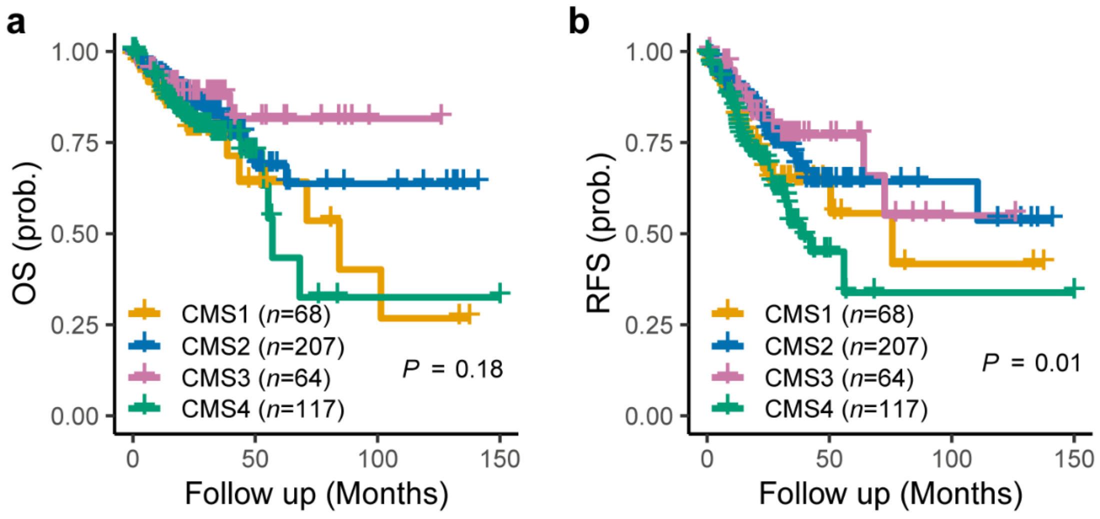

Supplementary Figure 2. Kaplan–Meier survival analysis showing the survival of CMS4 and other CMS subtypes. The CMS4 subtype presented worse overall survival (a) and recurrence-free survival (b) compared to other CMSs in the TCGA dataset.

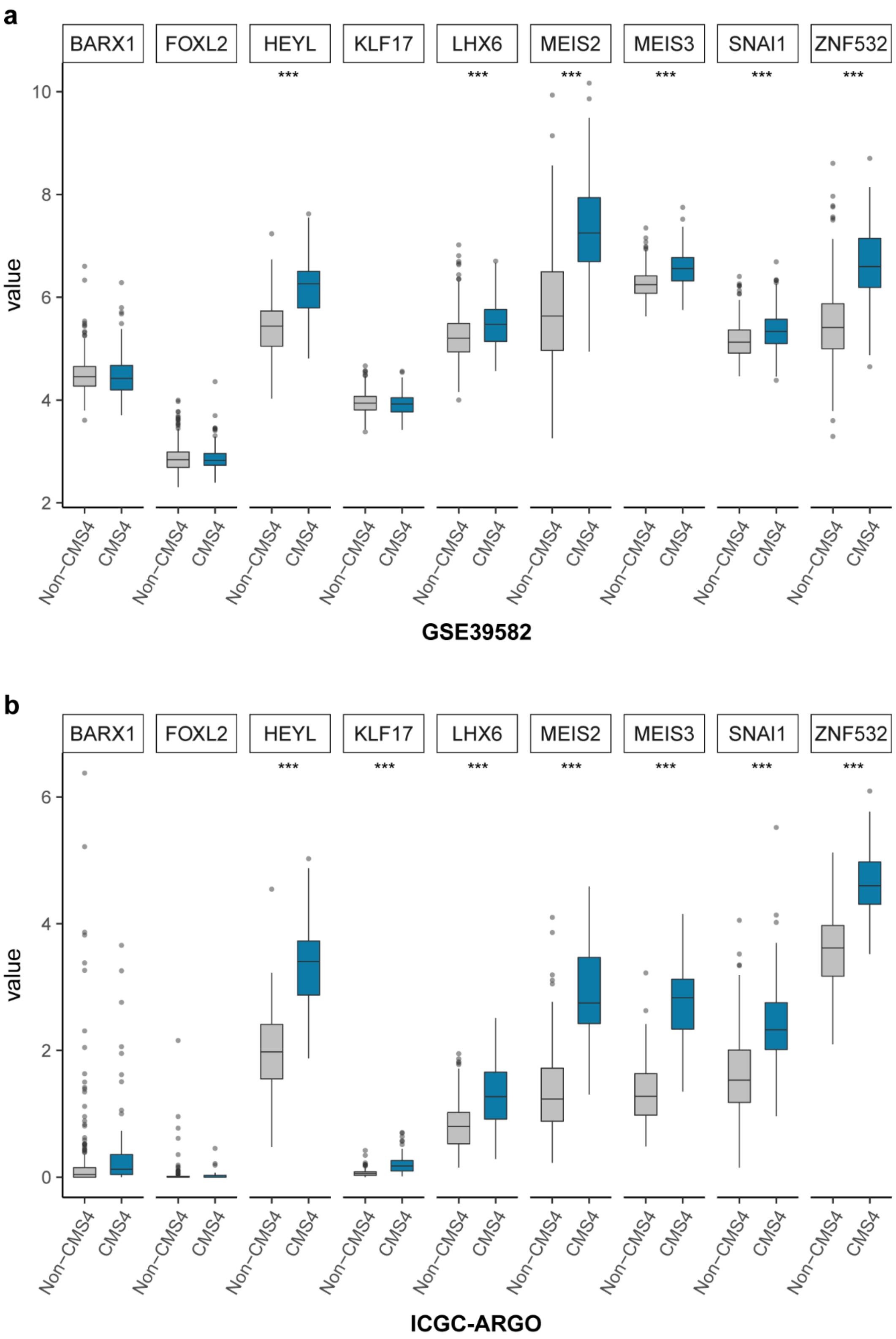

Supplementary Figure 3. The expression profile of all nine transcription factor genes in CMS4 and other CMS subtypes. Compared with other CMS subtypes, these nine candidate transcription factor genes were significantly up-regulated in the CMS4 subtype in GSE39582 (a) and ICGC-ARGO (b).

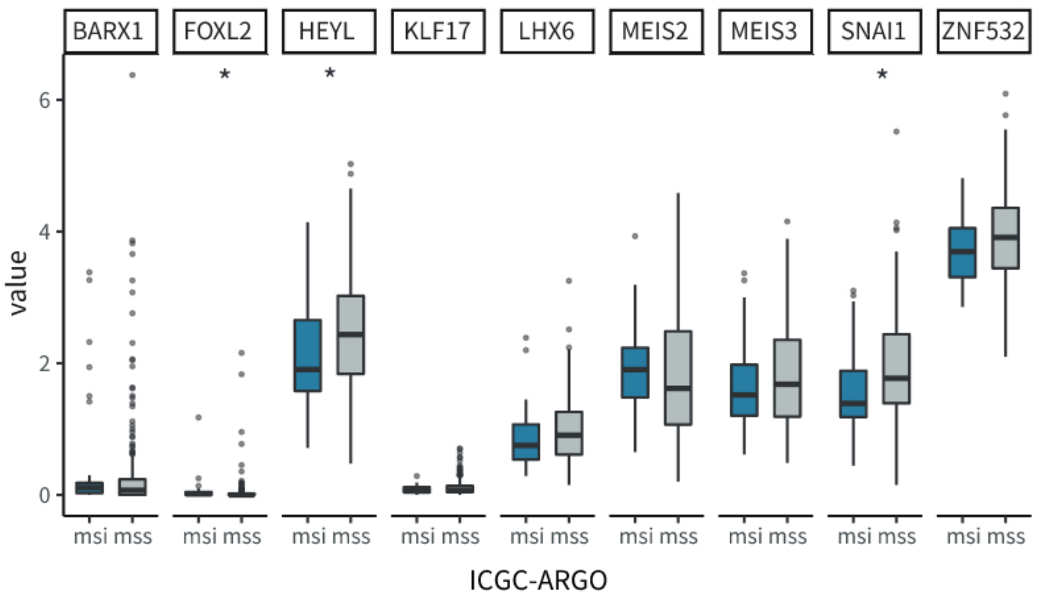

Supplementary Figure 4. The expression profile of all nine transcription factor genes in different MSI status. According to the microsatellite instability (MSI) status, HEYL and SNAI1 were down-regulated in MSI patients, FOXL2 was up-regulated, and the other six genes were not significantly different. (Supplementary Figure 4).

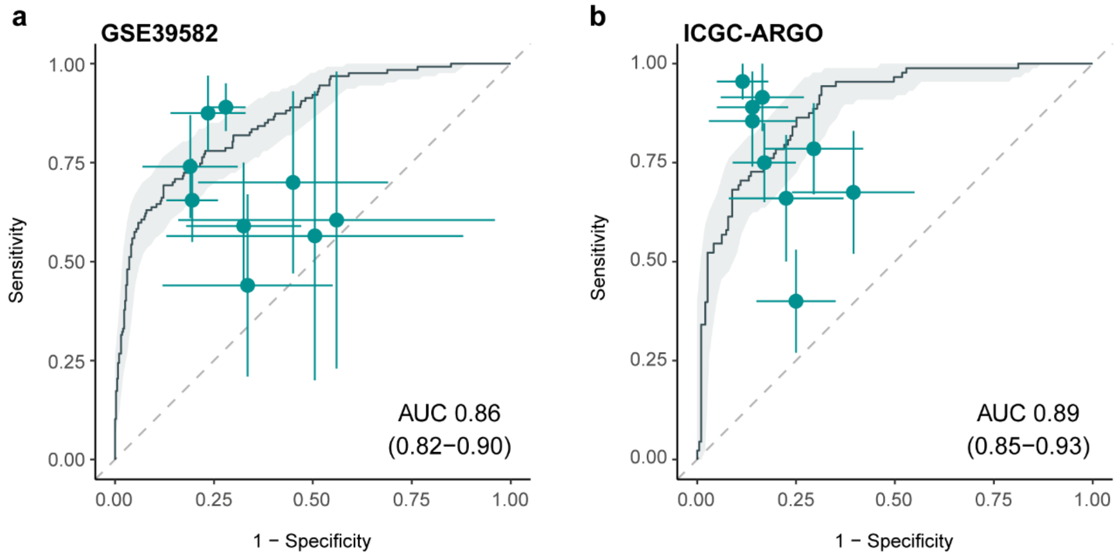

Supplementary Figure 5. The performance of TF-9 in identifying CMS4. TF-9 can distinguish CMS4 from other CMS subtypes of colorectal cancer, with AUC of 0.86 in GSE39582 (a) and AUC of 0.89 in ICGC-ARGO (b).

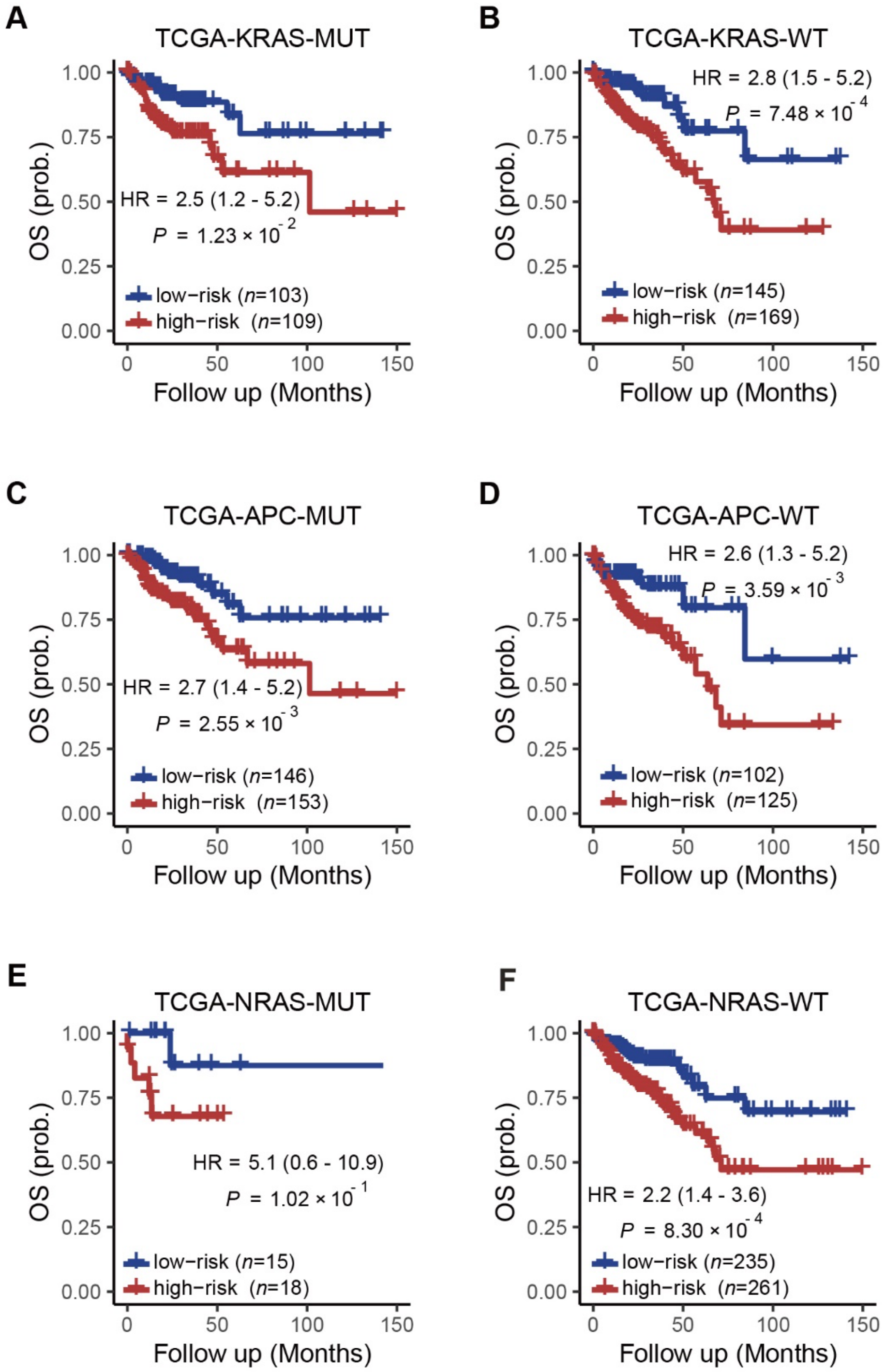

Supplementary Figure 6. The prognostic value of the TF-9 in colorectal cancer is stratified by mutation of RAS and APC. Even stratified by mutation of KRAS(A-B), APC(C-D), or NRAS(E-F), TF-9 can still stratify patients into low- and high-risk groups with significant prognosis values.

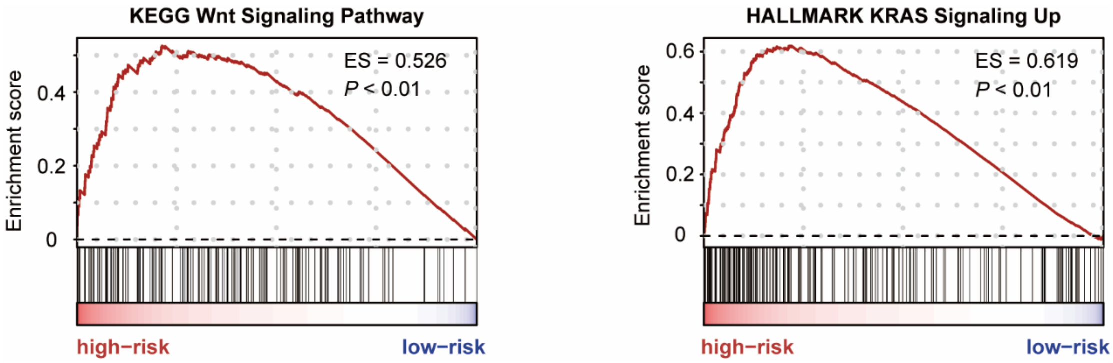

Supplementary Figure 7. The enriched pathways associated with TF-9 signature. GSEA revealed that these nine transcription factor genes are related to Wnt signaling pathway and KRAS signaling up.

Supplemental Table 1. Clinical and pathologic characteristics of all datasets

Supplemental Table 2. Univariate and multivariate analysis of TF signature and clinicopathological factors.

Supplemental Table 3. Master regulated analysis results

Supplemental Table 4. Patients risk stratification

Supplemental Table 5. GSEA results for the comparison of high- vs. low-risk groups

Supplementary Tables

Supplementary Table 1. Clinical and pathologic characteristics of all datasets

	Training cohort	Validation cohorts	
	TCGA (*n*=621)	ICGC-ARGO (*n*=351)	GSE39582 (*n*=565)
**Age (years)**	66 (31-90)	62 (25-93)	67 (22-97)
**Gender**			
female	290 (47%)	153 (44%)	255 (45%)
male	331 (53%)	198 (56%)	310 (55%)
**Location**			
left	351 (57%)	162 (46%)	341 (60%)
right	270 (43%)	119 (34%)	224 (40%)
unknown		70 (20%)	
**Stage**			
I	105 (17%)	31 (9%)	32 (6%)
II	229 (37%)	118 (34%)	264 (47%)
III	179 (29%)	90 (26%)	206 (36%)
IV	88 (14%)	112 (32%)	60 (11%)
unknown	20 (3%)		4 (1%)
**MSI status**			
MSI	188 (30%)	36 (10%)	74 (13%)
MSS	430 (69%)	296 (84%)	444 (78%)
unknown		19 (5%)	47 (8%)
**BRAF**			
wild		122 (35%)	460 (81%)
mut		10 (3%)	51 (9%)
unknown		219 (62%)	54 (10%)
**Subtype**			
CMS1	68 (11%)	39 (11%)	91 (16%)
CMS2	207 (33%)	96 (27%)	232 (41%)
CMS3	64 (10%)	56 (16%)	69 (12%)
CMS4	117 (19%)	88 (25%)	126 (22%)
unknown	165 (27%)	72 (21%)	47 (8%)
Median RFS, months (±SE)	19 (±0.96)	57 (±1.44)	43 (±1.71)
Median OS, months (±SE)	22 (±1.01)	60 (±1.31)	51 (±1.63)

Supplementary Table 2. Univariate and multivariate analysis of TF signature and clinicopathological factors.

	**Pooled validation cohorts**			
	**Univariate**		**Multivariate**	
	**HR (95% CI)**	***P***	**HR (95% CI)**	***P***
Gender (male vs. female)	1.35 (1.07-1.71)	0.01	1.42 (1.10-1.83)	0.006
Location (left vs. right)	0.87 (0.69-1.11)	0.26	0.73 (0.57-0.94)	0.010
MSI (MSI vs. MSS)	1.64 (1.07-2.51)	1.00E-03	1.70 (1.08-2.71)	0.020
Stage (I&II vs. III&IV)	2.56 (2.01-3.26)	2.9E-14	2.27 (1.74-2.98)	2.2E-09
Panel (high vs. low risk)	1.88 (1.49-2.38)	**1.3E-07**	1.72 (1.33-2.21)	**3.3E-05**
